# Interactive Improvements of Visual and Auditory Function for Enhancing Performance in Youth Soccer Players

**DOI:** 10.3390/ijerph16244909

**Published:** 2019-12-05

**Authors:** Young Hoon Song, Soo-Min Ha, Jang Soo Yook, Min-Seong Ha

**Affiliations:** 1 Department of Physical Education, Seoul National University, Gwanak-ro1, Gwanak-gu, Seoul 08826, Korea; canon0201@snu.ac.kr; 2 Laboratory of Exercise Physiology, Department of Physical Education, Pusan National University, 2 Busandaehak-ro 63beon-gil, Geumjeong-gu, Busan 46241, Korea; fantasista@pusan.ac.kr; 3 Center for Functional Connectomics, KIST Brain Research Institute, Korea Institute of Science and Technology 5, Hwarang-ro 14-gil, Seongbuk-gu, Seoul 02792, Korea; soulyook84@gmail.com; 4 Department of Sports Neuroscience, Advanced Research Initiative for Human High Performance (ARIHHP), Faculty of Health and Sport Sciences, University of Tsukuba, 1-1-1 Tennoudai, Tsukuba, Ibaraki 305-8574, Japan; 5 Laboratory of Exercise Biochemistry and Neuroendocrinology, Faculty of Health and Sports Sciences, University of Tsukuba, 1-1-1 Tennoudai, Tsukuba, Ibaraki 305-8574, Japan

**Keywords:** talented players, training, physical fitness, visual function, auditory function

## Abstract

We analyzed the effects of a regular training program on the health- and skill-related physical fitness (PF) of talented soccer players aged < 12 years; visual reaction time (VRT) and auditory reaction time (ART) were also assessed. In this single-group interventional study, 78 talented male youth soccer players (mean age, 9.54 years) were critically selected by the Korean Educational Development Institute and underwent a 22-week training program consisting of 16 weeks of PF and basic skill training (90 min/week) and 6 weeks of intensive training (3, 150-min sessions/week). We assessed the pre- and post-training body composition, cardiovascular endurance, muscle strength and endurance, and flexibility. We also measured power, agility, coordination and speed, passing ability, VRT, and ART. All variables improved after training. Post-training VRT correlated with ART, muscle mass, power, cardiovascular endurance, 10-m dribble time, 10-m ball touch count, and 10-m successful pass count. ART only correlated with muscle mass. ART and 10-m ball-touch count influenced VRT, and VRT influenced ART. In conclusion, the training program enhanced the PF and visual- and auditory-related reactions in talented youth soccer players. This study suggests the importance of the assessed relationships, indicating that a training program that improves these parameters enhances the players’ performance.

## 1. Introduction

Soccer is the most popular sport worldwide and is played by athletes of all ages. Globally, most regular leagues begin with youth leagues, and the number of players and leagues continues to grow. Previous studies have reported the benefits of the bilateral effects of sports specialization at an early age. Sports specialization with overtraining at a young age may have a negative influence on athletic performance due to burnout or increased risk of injury [1,2]. On the other hand, sports specialization with the appropriate training protocols and correct timing may be an effective strategy for achieving successful athletic performance [3]. In the context of youth sports, “talent” refers to a youth player with the potential to become a successful senior athlete in their specialized field [4]. Recently, research on talent identification and development of talented youth players in several sports, particularly in soccer, has increased [5,6]. This implies that the selected players are provided with a suitable training and learning environment to realize their potential. Hence, there is a need for talented youth player management, including the implementation of specific training programs.

In contemporary soccer, every player must be multi-functional and able to switch from playing a fast attack position to assuming a defensive position. The physical characteristics of the players have to be maintained because in soccer, physical factors and performance ability are very closely related. Players with better fitness can more successfully perform the necessary techniques and strategies and are less likely to experience injuries during play [7,8,9]. Performance research investigating the physical fitness (PF) of soccer players has shown varying results, depending on the player age, sex, and skills. Regardless, optimal PF, in accordance with the general requirements of the game, is important for all soccer players [10,11]. In the case of maturing youth soccer players, training results in significant physical performance improvements [12,13,14], and individualized training load has a great effect on aerobic fitness changes [15]. Among physical parameters, skill-related PF differs between elite/semi-elite players and “normal” people. Among the physical parameters, passing and dribbling skills are the best elements on which to determine a player’s performance level [16,17]. In particular, the dribbling skills of young players (13–16 years old) are clearly distinguished by their playing level [18]. Therefore, PF parameters can be divided into those that are health-related and those that are skill-related. The evaluation of skill-related PF requires the measurement of specific skill sets, including 10-m dribble time, ball touch count, and successful pass rate, among others.

Because the position of teammates and opponents changes constantly during a game, a player must be able to quickly predict and respond to rapidly changing situations. On-field changes require the player to make clear, quick, and effective decisions and apply their planned actions in a way that is cognitive; specifically, inhibitory control is needed [19]. This ability can be expressed as reaction function (RF), defined as the elapsed time between behavioral reactions following a sensory stimulus, which has been widely used as an indicator for the function of the sensory-motor system [20]. The visual component of reaction time (RT) is faster than the auditory component during exercise [21]. Elite youth soccer players have better control, cognitive flexibility, and metacognitive abilities [22], as well as better stop signal RTs, than amateur soccer players. As a result, they demonstrate superior suppression ability and lower error rates. In addition, RTs vary according to player position. Midfielders show faster auditory reaction times (ARTs) than do defenders and significantly faster visual reaction times (VRTs) than do strikers [23]. The importance of reaction training is emphasized because the RF and cognitive flexibility of young players is proportional to their competence [24]. Previous studies have mostly focused on independent changes, such as PF development and RT changes, after training [10,11,23,25]. However, there is a need to subdivide and analyze PF parameters and to closely analyze the relationship between PF and RF.

Given the importance of talent identification and development [5,6], in this study we applied a specific training program to youth soccer players < 12 years old who were selected through the talent development program of the Korean Educational Development Institute (KEDI). The study objective was to mix physiological and sensory-motor function perspectives when identifying the impact of and the relationship among PF (health- and skill-related variables), effect, and RF. We hypothesized that all variables would improve after a specific training program for talented youth soccer players, that there would be a relationship between PF parameters and RF, and that PF parameters—especially skill-related parameters—would affect RT.

## 2. Materials and Methods 

### 2.1. Participants

The required number of participants was calculated using the G*Power 3.1 sample counting program (Kiel University, Kiel, Germany). The results gave a significance level of 0.05, 70% power, and an effect size of 0.25, the default level in the paired *t*-test. Power analysis indicated that 77 participants were needed for a one-sided test; a total of 100 players were deemed to be an appropriate recruitment target, assuming that some would drop out [26,27]. Based on the evidence set out above, we initially selected 100 male talented youth soccer players, aged 7–11 years, from the youth academy of a professional Korean soccer club by the KEDI [28]. The initial recruitment of 100 talented players was made after the first criterion of document screening, the second criterion of interview, and soccer skill test by the KEDI. The physical characteristics of the participants are shown in Table 1. The selected players participated in all training programs and matches and did not perform any personal training other than team training. The participants did not have any injuries or neurological or musculoskeletal diseases that could affect the experiment; all parameters that could affect the experiment were controlled by doctor examinations/interviews and physical examinations. Before starting the study, the subjects were fully informed of the intent and purpose of the study, and parental consent and the consent of the participants were collected prior to the start of the experiment. The Institutional Human Research Committee of Seoul National University approved the research proposal (IRB #1610/001-003), which followed the Helsinki Declaration Guidelines and Ethical Principles.

### 2.2. Study Design

**Figure 1:**
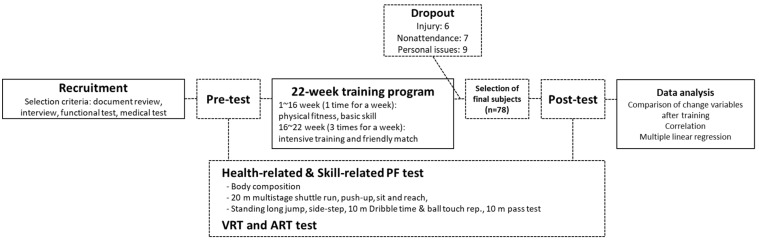
Overview of single group intervention study design. ART: auditory reaction time; PF: physical fitness; VRT: visual reaction time.

Based on a previous study demonstrating that a 22-week training period has beneficial effects on dribbling and passing skills in youth male soccer players [29], this 24-week study was conducted as a single group intervention study design for talented youth soccer players. It consisted of 1-week pre-test, a 22-week training session, and 1-week post-test (Figure 1). During the 22-week training program, 78 subjects were selected; 22 dropped out due to injuries or nonattendance. Pre- and post-tests were conducted before and after the 22-week training program, respectively. The measured test parameters were health-related PF, including body composition (height, weight, BMI, muscle mass, and fat mass), and cardiovascular endurance (20-m shuttle run). In addition, we measured strength (standing long jump), agility (side-step), coordination and speed (dribble test), and pass test as the skill-related PF parameters. We performed the above measures based on the released report from the Institute of Medicine program unit of the National Academies [30]. We also checked RF (visual and auditory reactions). All test parameters except RF were outstanding after two measurements. In the case of RF, the participants were instructed to practice three times in order to be familiarized with the visual and auditory selection tasks before entering the pre-test. Then, the test was conducted ten times for each visual and auditory reaction, and a one-minute break was given between visual and auditory tests. For the successful completion of this study, we thoroughly explained the purpose of the experiment to the subjects and monitored not only the training program, but also their lifestyle habits (eating habits, additional exercise, medical care, etc.) in real time.

### 2.3. Procedures

All test parameters were measured twice with the same method and conditions before and after the training program.

#### 2.3.1. Health-Related PF

##### (1). Body Composition

An X-Scan Plus II instrument (Jawon Medical, Seoul, Korea) was used to measure height, body weight, body mass index, muscle mass, and body fat mass [30]. Each participant had his height and weight measured automatically while comfortably standing with his feet slightly apart on the instrument and wearing simple clothing.

##### (2). Cardiovascular Endurance (20-m Shuttle Run)

The 20-m shuttle run, a cardiovascular endurance test [30], was measured in 20-m intervals at an initial speed of 8 km/h and measured using a sound source set to incrementally increase the signal interval by 0.5 km/h every minute. The participant started when a tone was heard and continued to run the 20-m interval while keeping up with the regularly accelerating audio rhythm. The test finished when the participant could not keep up with the audio rhythm. The times of the completed 20-m intervals were recorded.

##### (3). Muscular Endurance (Push-Up)

Push-ups were performed to measure muscular endurance [30]. With both hands shoulder-width apart on a 30-cm push-up bar, the arms were held in a straight line, perpendicular to the floor, and the feet together. The player bent his arms until his chest was within 5 cm of the bar while keeping his body straight, then straightened his arms again; this comprised one push-up. The number of push-ups completed within 2 min was recorded.

##### (4). Flexibility (Sit and Reach)

To measure flexibility [30], the participant sat on a sit-and-reach instrument and slowly bent the upper body to reach beyond the markings on the measuring instrument. After the movement of the fingertips had stopped for about 2 s, the distance reached was read automatically. The greatest distance out of two attempts was recorded.

#### 2.3.2. Skill-Related PF

##### (1). Power (Standing Long Jump)

To measure power [30], a standing long jump—also known as the horizontal jump—was performed because a previous study suggested that soccer players had greater leg asymmetries in the horizontal jump compared to the vertical jump [25,31]. Each participant stood at the top of a take-off board, without stepping on the line, and jumped as far as possible, with appropriate upper body movement. The landing point nearest to the take-off board was measured, and the best of two results was recorded.

##### (2). Agility (Side-Step)

A side-step test was performed to measure agility [30]. Using a side-step measuring instrument, each participant stood with his feet spread 1-m apart on either side of a center line. When indicated, he stepped to the right and left, with one foot stepping on or over the left or right line. The number of steps performed in 20 s was recorded.

##### (3). Coordination and Speed (Dribble Test)

A dribble test was used to simultaneously measure coordination and speed [30]. A 10-m dribble time was measured to identify coordination, intra-limb coordination, and speed. In addition, limb coordination and eye–foot coordination were measured by determining the number of times the ball was touched during the 10-m dribbling test. The 10-m dribble was performed twice and the average value was recorded [29].

##### (4). Accuracy and Consistency (Pass Test)

To measure passing accuracy and consistency, a 10-m pass test was performed to measure the accuracy and distance of the pass. The number of successful passes to a 0.5-m target 10 m away, out of 10 attempts, was recorded [29].

#### 2.3.3. RF (VRT and ART)

**Figure 2:**
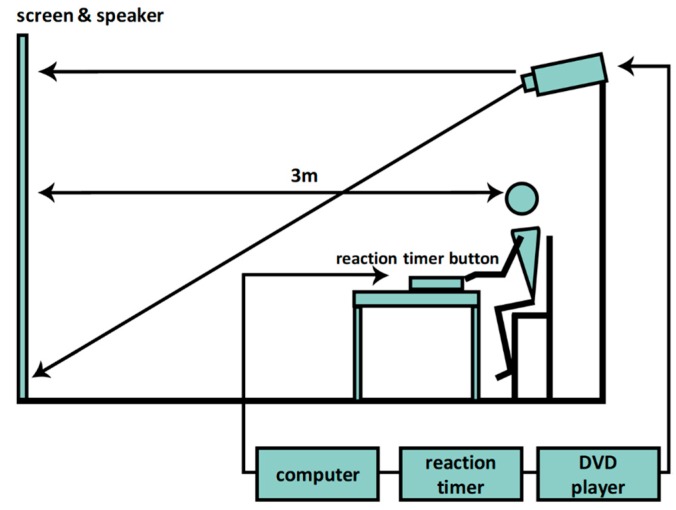
Experimental setup of reaction function. Note: Subjects were asked to press the button as quickly and accurately as possible following a random sequence of lights (blue, red, yellow) and sounds (500, 1000, 3000 Hz) while sitting on a chair with a reaction timer installed 3 m away from the screen.

A reaction timer (YB-1A, Japan) was used to evaluate VRT and ART. RT was measured as the time taken to press a button following a visual (blue, red, and yellow lights) or auditory (tones at 500, 1000, and 3000 Hz) signals (Figure 2). Each test consisted of three exercises and ten experiments. The players were asked to watch for the lights or listen for the tones, then to press the button as quickly and accurately as possible. The exercise that resulted in the quickest and most accurate reactions was chosen [20,25].

### 2.4. Training Program

The training program in this study cultivates youth talent to strengthen the competitiveness of domestic soccer by performing a talent search. The goal of the specific training protocol provided by the talent development program of the KEDI was to improve soccer talent and growth potential. The specific contents of the program include basic PF, coordination, basic training through physical and basic skills training once a week for 90 min from March to June (16 weeks), with customized training according to the growth rate of the child, and an intensive training program of three 150-min sessions a week from July to August (6 weeks). The 16-week specific training program is shown in (Table S1). During the 6 weeks of the intensive training session, evaluation games including soccer skill training and tactical training were conducted. We focused on the understanding of soccer as a team sport, such as positioning and tactical trend as well as tactical education, cooperation, and responsibility through evaluation games (Table S2). In addition, the players were able to gain a sense of game intuition through the matches, and they were constantly monitored for the soccer talent search.

### 2.5. Data Processing

The means and standard deviations of the measurement parameters were calculated using SPSS, version 20.0 (IBM, Armonk, NY, USA). For comparisons of the mean differences between the parameters, before and after the training program, were used the paired *t*-test. To determine correlations between the visual and auditory reactions and each parameter, normally distributed variables (based on a normality test) were analyzed using Pearson correlations; non-normally distributed variables were analyzed using Spearman rank correlations. Finally, we used stepwise multiple regression to analyze the impact of each variable on VRT and ART. Statistical significance was set at *p* < 0.05.

## 3. Results

### 3.1. Changes in Health-Related Fitness Following the 22-Week Soccer Training Program

**Figure 3:**
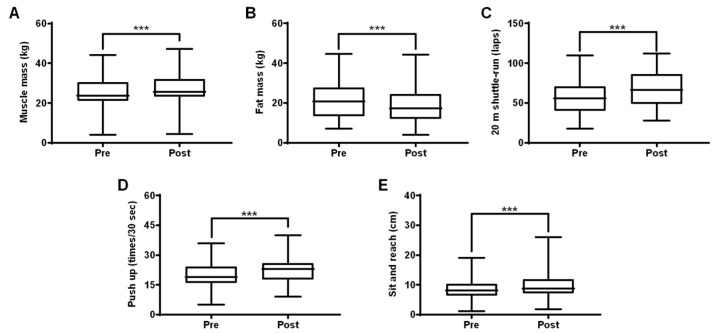
Effect of the training program on health-related physical fitness in talented youth soccer players. Positive changes in all health-related physical fitness parameters including muscle mass (**A**), fat mass (**B**), 20-m shuttle run (**C**), push-up (**D**), and sit and reach (**E**) of youth players after 22 weeks of training (*p* < 0.001). All values are presented as mean ± SD (*N* = 78). Note: *** *p* < 0.001 vs. pre-training (paired *t*-test).

We measured changes in health-related PF parameters, including body composition, cardiovascular endurance, muscular endurance, and flexibility, before and after the 22-week training program. All post-training health-related PF parameters were significantly improved (all *p* < 0.001). Muscle mass increased by 7.55%, while fat mass decreased by 11.26%. Cardiovascular endurance increased by 22.33%, muscular endurance increased by 16.29%, and flexibility increased by 21.98% (Figure 3, Table S3). These positive changes in health-related fitness demonstrate the effectiveness of the training program.

### 3.2. Changes in Skill-Related PF Following the 22-Week Soccer Training Program

**Figure 4:**
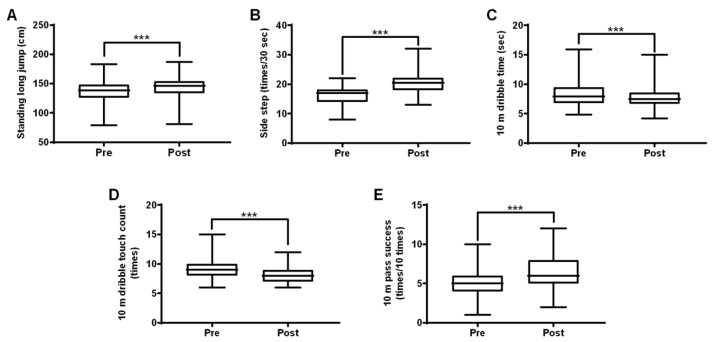
Effect of the training program on skill-related physical fitness in talented youth soccer players. Clear positive changes in all skill-related physical fitness parameters including standing long jump (**A**), side-step (**B**), 10-m dribble (**C**), 10-m dribble touch count (**D**), and 10-pass success (**E**) of youth players after 22 weeks of training (A, B, C, D and E: *p* < 0.001). All values are presented as mean ± SD (*N* = 78). Note: **p < 0.01, ***p < 0.001 vs. pre-training (paired *t*-test).

The changes in skill-related fitness after the 22-week training program are shown in Figure 4 and Table S4. After the training, power had increased by 7.55% (*p* < 0.001) and agility had increased by 28.84% (*p* < 0.001). The 10-m dribble time had decreased by 4.39% (*p* < 0.001) and the ball touch count had decreased by 4.27% (*p* < 0.001), indicative of coordination and agility improvements. The 10-m pass success count had also increased by 23.76% (*p* < 0.001), suggesting improvements in accuracy and consistency. Thus, the talented youth-specific training was effective for improving skill-related fitness.

### 3.3. Effects of RF Following the 22-Week Soccer Training Program

**Figure 5:**
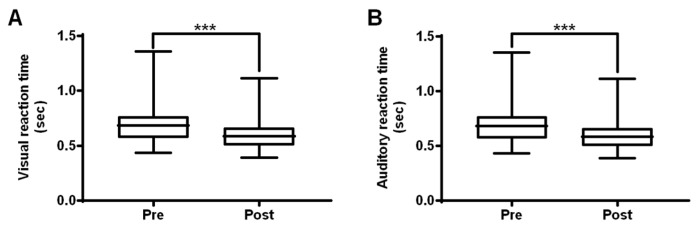
Effect of the training program on reaction function in talented youth soccer players. Reaction function parameters, including visual reaction time (**A**) and auditory reaction time (**B**) of youth players decreased significantly after 22 weeks of training (*p* < 0.001). All values are presented as mean ± SD (*N* = 78). Note: ***p < 0.001 vs. pre-training (paired *t*-test).

We measured VRT and ART to assess the impact of the training on player reaction rates. Post-training, VRT had decreased by 11.55% (*p* < 0.001) and the ART had decreased by 10.76% (*p* < 0.001). These results demonstrate the positive effects of the training program on participants (Figure 5, Table S5).

### 3.4. Correlations between VRT and ART and PF

We analyzed the correlations between VRT and ART and health-related and skill-related PF. First, we analyzed the correlations between VRT and other parameters pre-training. The higher the level of muscle mass (*r* = −0.522, *p* < 0.001), the power (*r* = −0.365, *p* < 0.01), agility (*r* = −0.315, *p* < 0.01), cardiovascular endurance (*r* = −0.236, *p* < 0.05), 10-m pass success count (*r* = −0.361, *p* < 0.01), 10-m dribble time (*r* = 0.333, *p* < 0.01), and dribble ball touch count (*r* = 0.474, *p* < 0.001), the faster the VRT. Besides, the faster the ART (*r* = 4.02, *p* < 0.001), the faster the VRT was found to be (Table S6).

**Figure 6:**
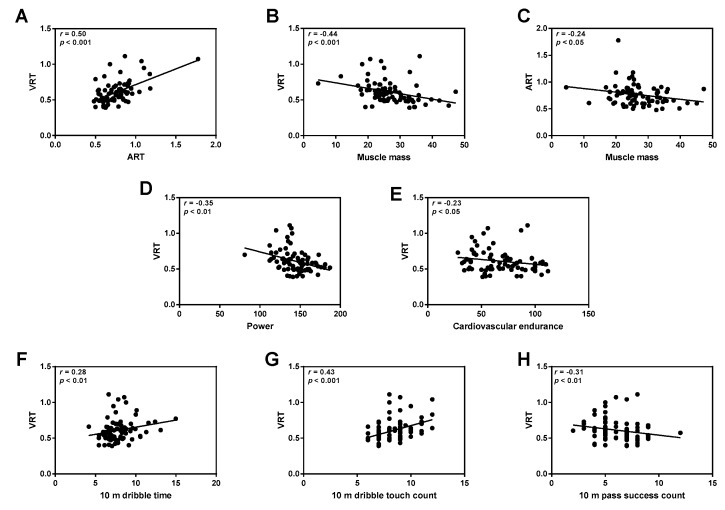
Correlation between reaction function (RF) and physical fitness (PF) parameters after training in talented youth soccer players. (**A**) The visual reaction time (VRT) significantly correlated with auditory reaction time (ART) (*r* = 0.50, *p* < 0.001). (**B**,**C**) Muscle mass significantly correlated with VRT (*r* = −0.44, *p* < 0.001) and ART (*r* = −0.24, *p* < 0.05). (**D**,**E**) The VRT positively correlated with power (*r* = −0.35, *p* < 0.01) and cardiovascular endurance (*r* = −0.23, *p* < 0.05). (**F**–**H**) VRT negatively correlated with 10-m dribble time (*r* = 0.28, *p* < 0.01) and 10-m dribble touch count (*r* = 0.43, *p* < 0.001) and positively correlated with 10-m pass success count (*r* = −0.31, *p* < 0.01). The black circles are the individual subjects’ (*N* = 78) levels (Pearson’s and Spearman correlation analysis was performed).

The post-training results showed that faster VRTs were also associated with increased muscle mass (*r* = −0.435, *p* < 0.01), power (*r* = −0.348, *p* < 0.01), cardiovascular endurance (*r* = −0.232, *p* < 0.05), 10-m pass success count (*r* = −0.313, *p* < 0.01), 10-m dribble time (*r* = 0.278, *p* < 0.01), 10-m touch count (*r* = 0.425, *p* < 0.001), and faster ARTs (*r* = 0.496, *p* < 0.001). ART improvement was correlated with the addition of muscle mass (*r* = −0.235, *p* < 0.05) (Figure 6).

### 3.5. Analysis of the Variables Influencing VRT and ART

We conducted a stepwise multiple regression analysis to determine which health- and skill-related PF variables had the greatest influence on VRT and ART; other independent variables were eliminated by stepwise selection. ART and 10-m ball touch count were identified as variables affecting VRT with an explanatory power of 43.8% (R^2^ = 0.438). The regression coefficient for ART was 0.424 and that for the 10-m ball touch count was 0.038 (*p* < 0.001), which were significantly higher than those for other variables (Table 2).

As a result of verifying the effect of variables on ART, VRT was identified as the parameter affecting ART, with an explanatory power of 30.4% (R^2^ = 0.304). The regression coefficient for VRT was 0.690 (*p* < 0.001), demonstrating a stronger effect than those for the other parameters (Table 3).

## 4. Discussion

To our knowledge, this is the first study to analyze physical parameters and RTs by combining physiological and motor control assessments in talented youth soccer players before and after their participation in a specialized training program. We demonstrated that the training program was effective at producing positive, health-related PF improvements in the participants as well as improving skill-related PF and RTs. VRTs and ARTs were also demonstrated to greatly influence each other; further, the number of times that a ball was touched during dribbling was found to influence VRT.

Humans perceive external conditions through the sensory nervous system and respond appropriately. RTs are reliable indicators of the rates at which sensory stimuli are processed and translated into motor reactions [32]. The ability to control motor responses is important in all sports, including soccer. A soccer player has to manage the ball as well as have fast RTs to manage changes. The RT level is an essential parameter for determining player competence [23], and professional players can control their motor reactions faster than amateur players [33]. Most previous studies investigating RTs have dealt with performance ability (elite player, non-elite player), proficiency, position, and prodigy selection [16,18,24]. Because we hypothesized that there would be a correlation between RT and PF variables, we aimed to identify the RTs of talented youth soccer players during their period of physical development plasticity and examine the PF variables related to ART.

In the present study, after 22 weeks of training, the players showed significant improvements in PF and RF variables compared to before the training. In general, the development of a mature athlete is accompanied by appropriate exercise at the right time. Children demonstrate nonlinear maturation, characterized by “developmental spikes” that affect the learning of specific athletic abilities at particular developmental stages [34]. Therefore, a properly timed training program will have a positive effect on health-related PF, skill-related PF, and RF. Thus, these were the expected outcomes of a long-term intensive training program.

This study demonstrated the relationship between post-training RTs, health-related PF, and skill-related PF. Interestingly, among the body composition parameters of health-related PF, VRT was most highly correlated with muscle mass, suggesting a relationship with cardiovascular endurance and skill-related PF parameters. Among the body composition parameters investigated, only muscle mass and fat mass were related to ART. VRT and ART are typical indicators of RF [35]. VRT is generally faster than ART during exercise [18] and VRT is more important than ART in soccer because it is essential for players to detect the movements of other players during a game [36]. The correlations observed in this study suggest that players with high muscle mass, agility, power, and the duration of these parameters, as well as those with developed dribbling and passing abilities, have high VRTs. ART is also related to body composition, but there is insufficient evidence to suggest the cause; therefore, additional ART research is required. The results of this study indicate that continuous monitoring of PF parameter changes is necessary when constructing training programs and that developing programs for improving muscle mass, power (squat jump and long jump), and balance are especially important [37]. The application of programs such as plyometric directional training, which has recently gained attention, are expected to be effective [38] and further improvements to the neuromuscular profile are needed to improve direction-specific muscular power [39]. In addition, skill-related PF parameters related to dribbling and passing will require ongoing training and education.

The focus of our study was on analyzing the health-related PF fitness and skill-related variables that had the most impact on VRT and ART. Although VRT and ART influenced each other, the 10-m ball-touch count was the variable determined to have the greatest impact on VRT. The correlation between VRT and ART was predictable based on correlations observed in previous studies. The present results suggest that a training program’s design, as well as its goals and direction, is important. In our training program, situational judgment and recognition, speed changes, and rhythmic dribbling were consistently emphasized through intensive dribbling training. Pass training was also emphasized, focusing on passing while considering defensive positioning and movements, passing while being conscious of the opponent, and controlling pass intensity. Finally, we believe that the design of the training program strengthened the understanding of each player’s position and that his ability to manage unpredictable situations played an important role in improving VRT due to improved dribbling, specifically the number of ball touches. 

This study observed long-term training effects on only talented youth athletes, and the results cannot be extended to non-talented subjects. However, our findings may be useful data because of the large number of participants. Although the study also revealed the training-induced beneficial effects on skill-related physical performance and visual and auditory function, there were limited outcome measurements. Using biomarker, neuroimaging, and physiological method approaches, further studies are needed to evaluate several limitations in the present study.

## 5. Practical Applications

Based on these results, players and coaches should recognize the importance of RT and PF on the field and should design training programs to improve these parameters and to improve player decision-making and performance in actual games. Sports scientists will need additional studies to analyze the relationship between various soccer skills as well as positions with RF.

## 6. Conclusions

A 22-week training program for talented soccer players under 12 years of age resulted in improved health-related PF, skill-related PF, and RF. Post-training VRT correlated with ART and PF parameters (muscle mass, power, cardiovascular endurance, 10-m dribble time, 10-m ball touch count, 10-m successful pass count); ART correlated with both VRT and muscle mass. In addition, the ART and 10-m ball touch count influenced VRT changes, and VRT had the strongest influence on ART changes.

## Figures and Tables

**Table 1:** Participant demographics.

Participants	Age (years)	Height (cm)	Weight (kg)	BMI (kg/m^2^)
Youth soccer player (*N* = 78)	9.54 ± 1.29	135.51 ± 9.72	33.11 ± 9.29	17.74 ± 3.13

BMI: body mass index. Values are mean ± SD.

**Table 2:** The influence of each variable on the post-training visual function (multiple regression: stepwise).

Variable	Dependent Variable	B	SE	*β*	*t*	DF	*F*
Auditory reaction time	Visual reaction time	0.424	0.069	0.530	6.114 ***	2	29.208 ***
10 m dribble touch		0.038	0.009	0.366	4.225 ***		

R = 0.662, R^2^ = 0.438, Adjusted R^2^ = 0.423. Note: *** *p* < 0.001.

**Table 3:** The influence of each variable on the post-training auditory function (multiple regression: stepwise).

Variable	Dependent Variable	B	SE	*β*	*t*	DF	*F*
Visual reaction time	Auditory reaction time	0.690	0.120	0.551	5.763 ***	1	33.206 ***

R = 0.551, R^2^ = 0.304, Adjusted R^2^ = 0.295. Note: *** *p* < 0.001.

## Supplementary Materials

The following are available online at https://www.mdpi.com/1660-4601/16/24/4909/s1, Table S1: Sixteen-week physical fitness and basic skills training program, Table S2: Six-week intensive training program, Table S3: Change of health-related physical fitness parameters after training, Table S4: Change of skill-related physical fitness parameters after training, Table S5: Change of reaction function after training, Table S6: Correlation between reaction function and other parameters pre and post training.
