# Supplementary material for: Interactive Improvements of Visual and Auditory Function for Enhancing Performance in Youth Soccer Players
Source: Int J Environ Res Public Health. 2019 Dec 5;16(24):4909. doi: 10.3390/ijerph16244909 (PMC6949993; doi:10.3390/ijerph16244909)
Supplement: Supplementary file 1 [file ijerph-16-04909-s001.pdf]

Table S1. 16-week physical fitness and basic skills training program.

No.	Training Topic	Instruction Detail
1	Ball Sense Training (inside, outside) Body Balance Training (coordination)	Acquiring soccer skills through ball sense training and ladder step training
2	Dribble Shooting (inside, outside) Body Balance Training (coordination)	Shooting after various dribbles Obtain a variety of technical skills through ladder step training
3	Practice Game	Instructive game activity with various techniques and cooperation in a given position
4	Dribbling Training	Develop dribbling ability to change direction
5	1:1 Shooting Drill	Cultivate ability to gain control over opponent through various dribble techniques and shooting training
6	Skill Training (ball control)	Understand ball control and develop ability to stop and keep the ball coming
7	Pass Practice (inside, outside)	Accurate delivery of ball to teammate standing still through inside pass and outside pass training
8	Friendly Match	Perform various techniques and cooperative programs according to given positions
9	Ball Sense Training (inside, outside, sole) Body Balance Training (coordination)	Acquire soccer skills through continuous ball sense training and ladder step training
10	Dribble Shooting (inside, outside, sole) Body Balance Training (coordination)	Use the insider and outside of the foot, but use the soles to learn how to shoot after dribbling
11	1:1, 2:2 Shooting Drills	Cultivate ability to gain control over opponent through various dribble techniques and shooting training
12	Friendly Match	Perform various techniques and cooperative programs according to given positions
13	Dribble Training Body Balance Training (coordination)	Obtain techniques to gain control over opponent through directional dribbling
14	Skill Training (ball movement control)	Understand ball control and improve movement control ability using various parts of the body
15	Pass Practice (inside, outside)	Develop ability to accurately pass to moving teammates
16	Friendly Match	Perform various techniques and cooperative programs according to given positions

Table S2. 6-week intensive training program.

Week	No.	Training Topic	Instruction Detail
1	1	Dribble Training I~II (switching direction & dribbling)	Flexible use of situation determination, change of dribble speed, etc.<br>Make necessary space in offense situation through dribbling
	2	Striker Movement Training (1:1)	Quickly dribble into empty space
	3	11 vs 11 Game	Understand position, induce competitiveness, motivation
2	4	Dribble Training I~II (dribbling & shooting)	Focus on accurate shooting by dribbling quickly in offense direction<br>Improve dribbling ability in case of competition
	5	Passing Game & Side Play	Strength-controlled coaching of passes<br>Set path target point after recognizing surrounding situation<br>2:1 pass coaching on the side
	6	11 vs 11 Game	Understand position, induce competitiveness, motivation
3	7	Dribble Training I~II (dribble & pass)	Rhythmic dribbling<br>Dribble & pass considering the defense position and movement (situation recognition)
	8	6 vs 5 Strategy Training and Total Drill	Timed pass to the player to the right<br>Second movement check for ball
	9	11 vs 11 Game	Understand position, induce competitiveness, motivation
4	10	Dribble Training I~II (technical dribbling & competitive situation offense)	Improve fast dribbling ability to recognize defense position and distance<br>Space invasion and shooting after offense direction dribbling
	11	1:1, 2:1, 2:2 Central Offense	Secure clear view by lifting your head so you can better understand the situation<br>Improve quick dribbling ability by recognizing the defense position and distance from the front<br>Speedy dribble to connect the shooter
	12	11 vs 11 Game	Understand position, induce competitiveness, motivation
5	13	1:1, 2:1 Side Offense	Speed change coaching when you move to deceive your opponent<br>Combination play in terms of overlap, underlap, etc.
	14	1:1 Individual Defense	Acceleration and deceleration maintaining proper distance and angle to target point<br>Step-by-step switching direction instruction
	15	11 vs 11 Game	Understand position, induce competitiveness, motivation
6	1	2:2 Cooperative Defense	Instruction on ball pressure and cover play<br>Predicting opponent's unstable ball touch and emphasis on active cooperative defense
	2	Strategy Training and Total Drill	Organic motion coaching according to ball flow<br>Positioning and speed change coaching
	3	11 vs 11 Game	Understand position, induce competitiveness, motivation

Table S3. Change of health-related physical fitness parameters after training.

Variable	Pre-training	Post-training	t
Muscle mass (kg)	25.10±6.46	26.92±6.99	-8.679***
Fat mass (kg)	21.30±9.12	18.53±8.45	5.205***
Cardiovascular endurance (laps)	57.79±20.69	67.71±22.90	-6.727***
Muscular endurance (times/30sec)	19.87±6.32	22.47±6.36	-8.503***
Flexibility (cm)	8.45±3.36	9.95±4.27	-6.547***

Paired t-test. Values are mean ± SD. Note: ***p < 0.001 vs pre-training.

Table S4. Change of skill-related physical fitness parameters after training.

Variable	Pre-training	Post-training	t
Power (cm)	137.06±19.34	144.18±18.43	-8.037***
Agility (times/30sec)	16.37±3.03	20.61±3.94	-9.611***
10 m Dribble time (sec)	8.42±2.42	7.85±1.83	3.569***
10 m Dribble touch (times)	8.89±1.62	8.44±1.49	3.490***
10 m Pass success count (times)	5.22±1.88	6.12±1.89	-6.596***

Paired t-test. Values are mean ± SD. Note: ***p < 0.001 vs pre-training.

Table S5. Change of reaction function after training.

Variable	Pre-training	Post-training	t
Visual reaction time (sec)	0.70±0.16	0.61±0.16	6.401***
Auditory reaction time (sec)	0.87±0.2	0.77±0.20	5.838***

Paired t-test. Values are mean ± SD. Note: ***p < 0.001 vs pre-training.

Table S6. Correlation between reaction function and other parameters pre and post training.

Variable	Visual reaction time		Auditory reaction time	
	Pre-training	Post-training	Pre-training	Post-training
Muscle mass	-0.522***	-0.435**	-0.220	-0.235*
Fat mass	-0.205	-0.165	-0.339**	-0.174
Muscular endurance	-0.205	-0.158	-0.001	-0.133
Power	-0.365**	-0.348**	-0.160	0.156
Flexibility	0.022	0.157	0.056	0.008
Agility	-0.315**	-0.142	-0.031	-0.011
Cardiovascular endurance	-0.236*	-0.232*	-0.092	-0.150
10 m Dribble time	0.333**	0.278**	0.012	0.064
10 m Dribble touch	0.474***	0.425***	0.038	0.057
10 m Pass success count	-0.361**	-0.313**	-0.017	-0.185
Auditory reaction time	0.402***	0.496***		

Note: *p < 0.05, **p < 0.01, ***p < 0.001.
